# Complete mitochondrial genome and the phylogenetic position of theTaiwan spurdog shark *Squalus formosus* (Squaliformes: Squalidae)

**DOI:** 10.1080/23802359.2016.1176884

**Published:** 2016-06-20

**Authors:** Hao Chen, Xiao Chen, Haibin Yu, Weiming Ai

**Affiliations:** a Department of Marine Biotechnology, School of Life Science, Wenzhou Medical University, Wenzhou, People’s Republic of China;; b Zhejiang Mariculture Research Institute, Wenzhou, People’s Republic of China

**Keywords:** Mitochondrial genome, Squalidae, *Squalus formosus*

## Abstract

The complete mitogenome of the Taiwan spurdog shark *Squalus formosus* (Squaliformes: Squalidae) is determined in this study. It is a circle molecular (16,735 bp), containing 37 genes with typical order to that of most other vertebrates. The nucleotide composition is: A 30.9%; C 24.5%; G 14.2%; T 30.5%. Two start codons (GTG and ATG) and two stop codons (TAG and TAA/T) are used in the protein-coding genes. 22 tRNA genes range from 66 bp (tRNA-*Ser*2) to 75 bp (tRNA-*Leu*1). The phylogenetic result shows that *S. formosus* clusters to the (*Squalus acanthias* +* Cirrhigaleus australis*) clade.

The Taiwan spurdog shark *Squalus formosus* (Squaliformes: Squalidae) is a new species in the genus *Squalus* of the highfin megalops group which is found and identified in recent years (White & Iglésias 2011). This species mainly habitats in Pacific Ocean (Taiwan and Japan). Although *S. formosus* is morphologically closest to *Squalus albifrons* from eastern Australia, it can be distinguished from other *Squalus* species in some morphological characters. Here in this study, we determine its complete mitochondrial genome and construct the Bayesian tree to find the phylogenetic position of *S. formosus.*

One specimen of *S. formosus* was captured from East China Sea and preserved in Wenzhou Medical University with voucher SF2012061405. The experimental protocol, data analysis and Bayesian phylogenetic reconstruction followed from the study of Chen et al. (2015). The outgroup *Chimaera monstrosa* and 15 species of Squalimorphs with complete mitogenomes available in the GenBank were selected to construct the phylogenetic tree.

The whole mitogenome of *S. formosus* is 16,735 bp in length (GenBank Accession Number: KU951280) with a normal overall base composition of the H-strand (A 30.9%; C 24.5%; G 14.2%; T 30.5%) and its gene composition, arrangement and transcriptional orientation is identical to most mitogenome of sharks. This circle molecular contains 37 genes (13 protein-coding genes, 22 tRNA genes and 2 rRNA genes) and one non-coding control region. There are total 33 bp short intergenic spaces located in 13 gene junctions and 24 bp overlaps located in 6 gene junctions in the whole mitogenome. Among protein-coding genes, one gene (*ND*6) located in light-strand, the remaining all in heavy-strand. Except for the *CO*1 gene starting with GTG codon, all remaining protein-coding genes used standard ATG codon as their initial codon. Except for the *ND*6 gene using TAG as its terminal codon, all others use typical TAA/T codon. 22 tRNA genes ranges from 66 bp (tRNA-*Ser*2) to 75 bp (tRNA-*Leu*1), all of which except for tRNA-*Ser*2 can fold into a typical clover-leaf secondary structure. The origin of L-strand replication (35 bp) is identified between tRNA-*Asn* and tRNA-*Cys* genes, which can fold a hairpin structure as a signal to initiate the replication of L-strand. Both 12S rRNA (951bp) and 16S rRNA (1676 bp) genes are between tRNA-*Phe* and tRNA-*Leu*1 genes, separated by tRNA-*Val* gene. The control region (1079 bp, rich in A + T content (66.9%)) is located between the tRNA-*Pro* and tRNA-*Phe* genes.

In the Bayesian tree, four orders are showed and most nodes within them are strongly supported (Figure 1). Three orders (Pristiophoriformes, Squatiniformes and Hexanchiformes) are monophyletic according to topology. The order Hexanchiformes is relatively basal and primitive. Within the family Squalidae of the order Squaliformes, *Squalus acanthias* clustered to *Cirrhigaleus australis*, then *S. formosus* clustered to this clade. In addition, within the family Dalatiidae, *Squaliolus aliae* and *Somniosus pacificus* clustered to the species of Etmopteridae and Suqalidae, respectively. It demonstrates that the genus *Squalus* and the family Dalatiidae are paraphyletic. Therefore, the relationship within the order Squaliformes needs further study.

**Figure 1.**
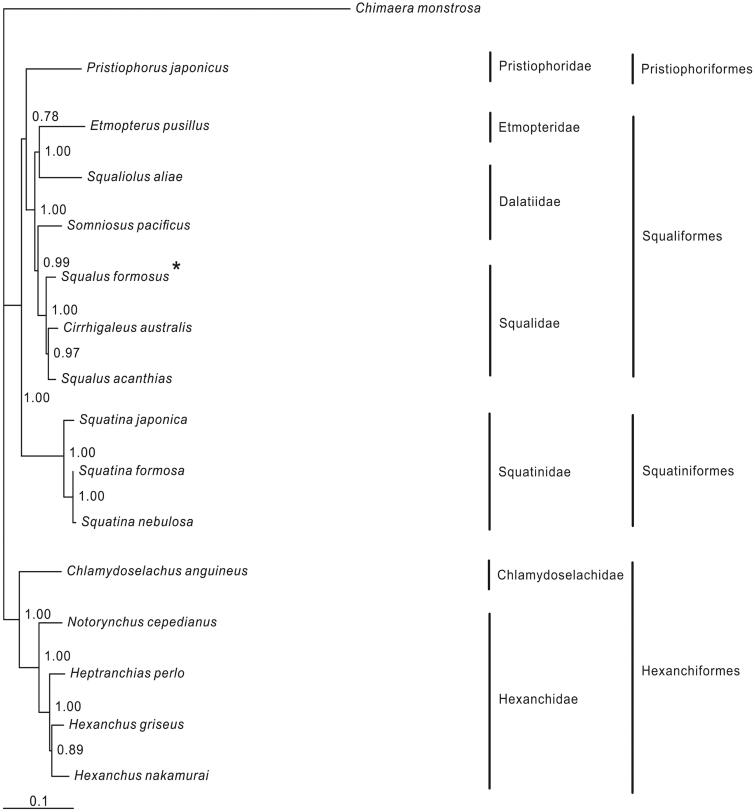
Phylogenetic position of *Squalus formosus*, *Chimaera monstrosa* (AJ310140.1) is selected as the outgroup. One species from the order Pristiophoriformes is: *Pristiophorus japonicus* (NC_024110.1). Five species from the order Squaliformes are: *Somniosus pacificus* (NC_022734.1), *Squaliolus aliae* (KU873080), *Etmopterus pusillus* (KU892588), *Cirrhigaleus australis* (KJ128289), *Squalus acanthias* (NC_002012.1), *S. formosus* (KU951280). Three species from the order Squatiniformes are: *Squatina japonica* (NC_024276), *S. nebulosa* (NC_025578.1), *S. formosa* (KM084865). Five species from the order Hexanchiformes are: *Chlamydoselachus anguineus* (NC_022729), *Heptranchias perlo* (NC_022730), *Hexanchus griseus* (KF894491), *H. nakamurai* (AB560491), *Notorynchus cepedianus* (NC_022731).
